# Integrated analysis of microRNA and mRNA expression profiles in abdominal adipose tissues in chickens

**DOI:** 10.1038/srep16132

**Published:** 2015-11-04

**Authors:** H. Y. Huang, R. R. Liu, G. P. Zhao, Q.H. Li, M. Q. Zheng, J. J. Zhang, S. F. Li, Z. Liang, J. Wen

**Affiliations:** 1 Institute of Animal Sciences, Chinese Academy of Agricultural Sciences, Beijing 100193, P.R. China; 2 Institute of Poultry Science, Chinese Academy of Agriculture Sciences, Yangzhou 225125, P. R. China

## Abstract

Excessive fat accretion is a crucial problem during broiler production. Abdominal fat weight (AbFW) and abdominal fat percentage (AbFP) are major phenotypic indices of fat traits. The present study used F2 females derived from a cross between Beijing-You and Cobb-Vantress chickens. Cohorts with extreme AbFP and AbFW phenotypes were chosen to construct high- and low-abdominal fat libraries (HAbF and LAbF, respectively) to investigate the expression profiles by RNA-sequencing and microRNA (miRNA)-sequencing. Compared with the LAbF library, 62 differentially expressed miRNAs (DEMs) and 303 differentially expressed genes (DEGs) were identified in the HAbF birds. Integrated analysis of DEMs and DEGs showed that a total of 106 DEGs were identified as target genes for the 62 DEMs. These genes were designated as intersection genes, and 11 of these genes are involved in lipid metabolism pathways. The miRNA gga-miR-19b-3p accelerated the proliferation of preadipocytes, as well as adipocyte differentiation, by down- regulating *ACSL1*. These findings suggest that some strong candidate miRNAs and genes, important in relation to abdominal adipose deposition, were identified by the integrated analysis of DEMs and DEGs. These findings add to our current understanding of the molecular genetic controls underlying abdominal adipose accumulation in chickens.

Decades of intensive genetic selection have resulted in increased body weight gain, growth rate, and feed conversion efficiency in broiler chickens^1^. Unintended effects include the accompanying excess in fat deposition, especially that of abdominal fat. Contemporary broiler breeds contain 150 to 200 g of fat per kg body weight, 85% of which is not physiologically essential and represents an inefficiency in meat production. For this reason, excessive fat accretion is a crucial problem in broiler production. Abdominal fat weight (AbFW) and abdominal fat percentage (AbFP) are major phenotypic indices of fat traits. Abdominal fat (AbF) reflects complex underlying genetic controls and has moderate heritability (h^2^ = 0.62 for AbFW, and 0.24 for AbFP)^2^. Therefore, it should be feasible to modulate AbF deposition and this goal will be aided by better understanding its molecular genetic controls.

MicroRNAs (miRNAs) are endogenous, non-coding small regulatory RNAs of 19 to 24 nucleotides (nt) that bind to complementary target sites in the 3′ untranslated region (UTR) of mRNAs, resulting in translational repression and/or mRNA destabilization^3^,^4^,^5^. Mature miRNAs are derived from pri-miRNA precursors composed of hundreds or thousands of nt that constitute monocistronic or polycistronic transcriptional units^6^,^7^,^8^. These small regulatory RNAs have been implicated in numerous and wide-ranging biological processes, including cell proliferation, differentiation, development, apoptosis, pathogenesis, disease resistance, tumorigenesis, and lipogenesis^9^,^10^,^11^,^12^,^13^,^14^,^15^. Chicken miRNAs in a variety of tissues have been identified recently with a combination of methods. Solexa sequencing identified microRNAs in somites of developing chicken embryos^16^, as well as differentially expressed miRNAs in sexually mature and immature chicken ovaries^17^. The 454 Life Sciences methodology identified hepatic miRNAs on embryo days 15, 18, and 20, and post-hatch days 0, 3, 7, and 14^18^. Additionally, 159 known miRNAs were detected in Arbor Acres pre-adipocytes (by Solexa sequencing), several of which (e.g., miR-222, miR-30d, miR-26a, let7c, let7a, and miR-30a-5p) were highly expressed^19^. In contrast, only 48 known miRNAs in chicken adipose tissue have been identified by cloning^14^, as only high abundance and known miRNAs have been identified. Novel miRNAs, as well as those of low abundance, have not been detected, and potentially important miRNAs could have been overlooked. It is essential to fully identify the miRNAs in abdominal fat by deep sequencing, and to establish the transcriptome of chicken abdominal adipose tissue. A time-course analysis of gene expression in the abdominal fat of male chickens from fat and lean lines during juvenile development (1 to 11 weeks of age) has been performed using the Del-Mar 14K Chicken Integrated Systems microarray^20^. The present study was undertaken to use integrated analyses of differentially expressed miRNAs (DEMs) and differentially expressed genes (DEGs), which have not been previously reported, to obtain a comprehensive view to reveal their functions in lipid metabolism and their related regulatory pathways.

The present study used F2 females derived from a cross between a slow-growing Chinese local breed (Beijing-You) and a rapid-growing commercial broiler line (Cobb-Vantress). Cohorts of birds with extreme AbFP and AbFW phenotypes were chosen to investigate the expression profiles of mRNAs and miRNAs by RNA-sequencing and miRNA-sequencing. The focus here was on a deep analysis of critical genes, miRNAs, and pathways using the miRNA and mRNA expression profiles related to abdominal fat deposition.

## Results

### miRNA expression profiling and screening of differentially expressed miRNAs

There were a total of 6,526,815 and 7,784,759 raw reads in the range of 16–32 nt in the two libraries (HAbF and LAbF), of which 3,503,932 (53.7% of raw reads) and 1,637,052 (32.5% of raw reads), respectively, were mappable and could be aligned to unique miRNAs. The size distribution of reads was not significantly different in the two libraries, and the majority of the reads had lengths of 21–24 nt (Figure S1).

Mappable reads were aligned to selected precursors and mature sequences in the miRBase database and further mapped to the chicken genome. As described in Table 1, mappable reads constituted three groups: Groups 1, 2, and 3. Unmappable reads were related to the Rfam/Repbase, mRNA database, and chicken genome to predict novel miRNA hairpin structures (Group 4) and other RNAs (e.g., rRNAs, tRNAs, mRNAs, etc.). Six hundred five unique sequences were identified on the basis of mature miRNA sequences in miRBase (release 19.0), including 230 known chicken miRNAs, and 33 miRNAs were mapped to the chicken genome in groups 1b and 2a. Eighty-three potentially novel miRNAs were identified and defined as PC-3p or PC-5p (Table S1).

miRNA expression profiles of the HAbF and LAbF pools were compared, and differentially expressed miRNAs (DEMs) were identified (*P* ≤ 0.05 by Fisher's exact test and the chi-square test, and fold-change ≥1.5 or ≤0.67) based on RPM ≥ 5 in either of the two groups. Compared with the LAbF library, 62 miRNAs were differentially expressed (32 up-regulated and 30 down-regulated), as detailed in Table S2.

**Figure 1:**
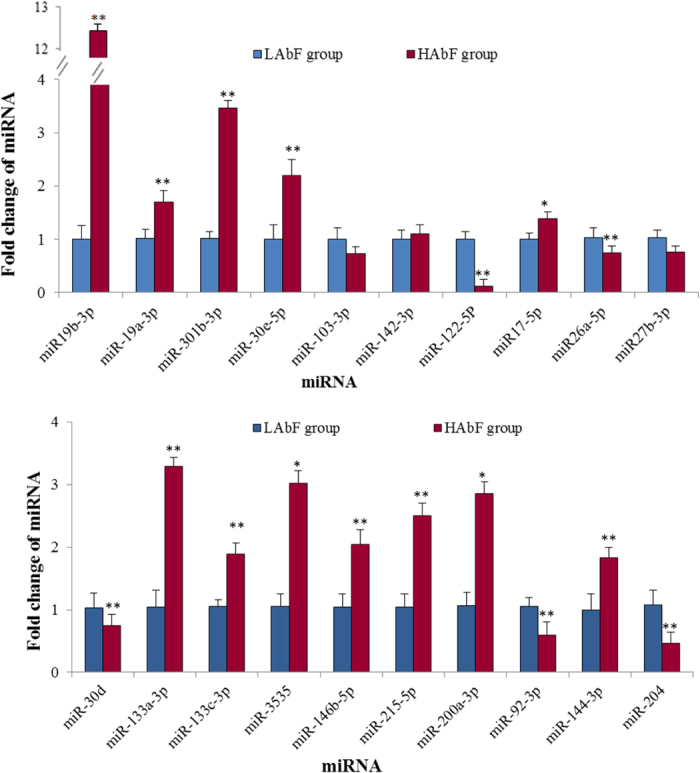
Validation of differentially expressed miRNAs by qPCR, *P < 0.05, **P < 0.01.

Using Beijing-You birds that comprised HAbF or LAbF extremes, quantitative real-time PCR (qPCR) was used to verify 20 of the DEMs identified by deep sequencing. Eleven of these (miR-204, miR-19a-3p, miR-19b-3p, miR-30d, miR-26a, miR-122-5p, miR-103-3p, miR-27b-3p, miR-92-3p, miR-142-3p, and miR-17-5p) have been implicated, directly or indirectly, in fat deposition; 9 showed a high fold-change (miR-3535, miR-144-3p, miR-30e-5p, miR-301b-3p, miR-215-5p, miR-200a-3p, miR-133a-3p, miR-133c-3p, and miR-146b-5p). With the exception of miR-204, miR-215-5p, miR-142-3p, and miR-103-3p, there was consistency between the qPCR assays and the deep sequencing analysis in terms of the direction of regulation and statistical significance (Fig. 1). Of particular note, there was a 12-fold up-regulation (five-fold in deep sequencing) of miR-19b-3p, and an eight-fold down regulation (six-fold in deep sequencing) of miR-122-5p between HAbF and LAbF chickens (*P* < 0.05).

### mRNA expression profiling and screening of differentially expressed genes

The content of identifiable mRNAs in the adipose transcriptomes of the HAbF and LAbF pools from F2 birds was assessed by deep sequencing (for details of quality control, see Table S3 and Figure S2). Of approximately 5790 transcripts detected, 430 differentially expressed genes (DEGs) were identified (*P* ≤ 0.05, fold-change ≥2 or ≤0.5) based on FPKM ≥ 10 in either of the two pools. Of the DEGs, 303 were known chicken genes (189 up-regulated and 114 down-regulated in HAbF). An additional 60 transcripts were unknown genes, and 67 transcripts were identified as novel transcripts (Table S4).

**Figure 2:**
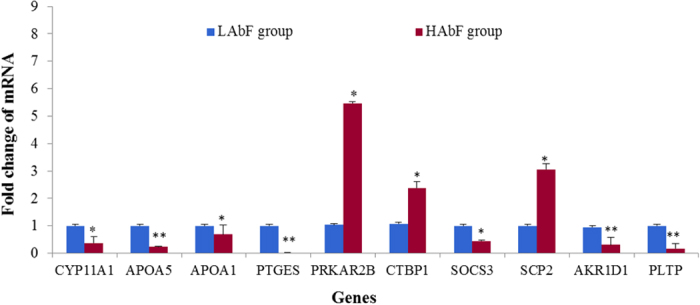
Validation of differentially expressed genes by qPCR, *P < 0.05, **P < 0.01.

The deep sequencing findings were verified by independent qPCRs of RNA isolated from birds with extreme AbF phenotypes. The expression of 10 genes (*CYP71A1*, *APOA5*, *APOA1*, *PTGES*, *PRKAR2B*, *CTBP1*, *SOCS3*, *SCP2*, *AKR1D1*, and *PLTP*) having a high fold-change by deep sequencing and/or known to have a direct or indirect association with abdominal fat showed consistency between the qPCR assays and the deep sequencing analysis in terms of the direction of regulation and statistical significance (Fig. 2).

### Integrated analysis of DEMs and DEGs

As described above, DEGs were identified on the basis of *P* ≤ 0.05 and fold-value ≥2.0 or ≤0.5, while DEMs were chosen using RPM ≥ 5 and fold-change ≥1.5 or ≤0.67. In this study, the target genes of the miRNAs were predicted on the basis of chicken sequences using the TargetScan^21^,^22^,^23^,^24^ (http://www.targetscan.org) and miRanda (http://cbio.mskcc.org/miRNA2003/miranda.html) algorithms. A total of 6121 target genes for the 62 DEMs were identified. Among these, 106 genes were found to be DEGs by mRNA sequencing analysis and were designated as “intersection genes” (Table S5).

Of the 86 intersection genes associated with the 31 up-regulated miRNAs, the expression of 71 genes was increased, while the expression of 15 genes was decreased in the HAbF birds compared with the LAbF birds. The majority (83/100) of the genes associated with the 31 down-regulated miRNAs showed increased abundances in HAbF birds, but the transcripts of 17 genes were down-regulated.

### Pathway and GO analysis for intersection genes

**Figure 3:**
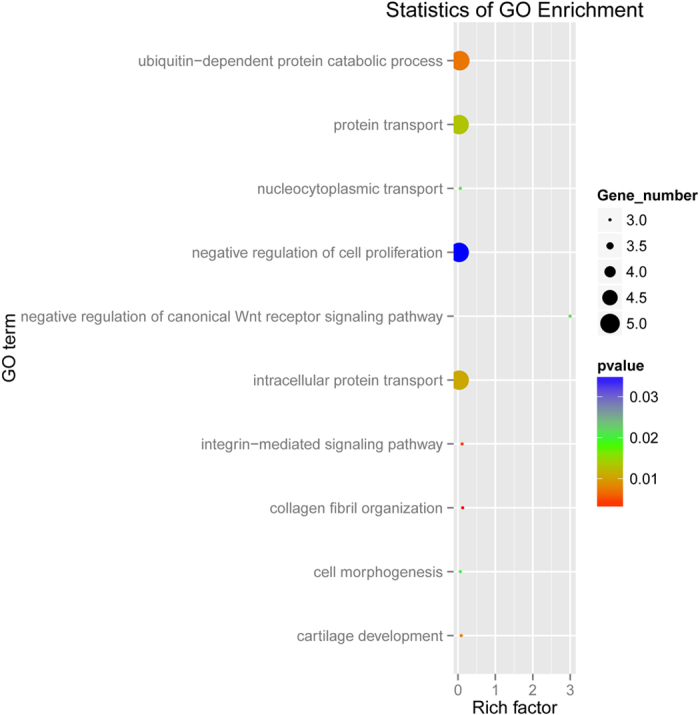
The significantly enriched GO terms. *P* ≤ 0.05.

The significantly enriched GO term based on the 106 intersection included collagen fibril organization, integrin-mediated signaling pathway, ubiquitin-dependent protein catabolic process, intracellular protein transport, nucleocytoplasmic transport, cell morphogenesis, negative regulation of canonical Wnt receptor signaling pathway and negative regulation of cell proliferation biological process (Fig. 3). The significantly enriched pathways (Table 2) included fatty acid metabolism, Biosynthesis of unsaturated fatty acids focal adhesion, peroxisome proliferator-activated receptor (PPAR) signaling, peroxisome, and ECM-receptor interaction. Several genes play roles in multiple pathways. For example, fatty acid desaturase 2 (*FADS2)* and stearoyl-CoA desaturase (*SCD*) are involved in the biosynthesis of unsaturated fatty acids, as well as in fatty acid metabolism and PPAR signaling, while peroxisomal trans-2-enoyl-CoA reductase (*PECR*) and acyl-CoA synthetase long-chain family member 1 (*ACSL1*) participate in the biosynthesis of unsaturated fatty acids, fatty acid metabolism, peroxisome, and metabolism pathways.

**Figure 4:**
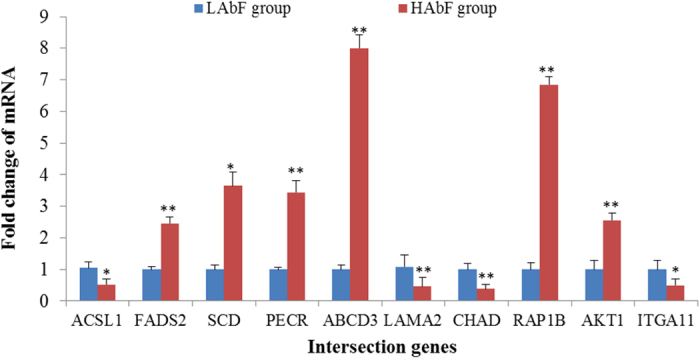
Validation of intersection genes by qPCR, *P < 0.05, **P < 0.01.

Changes in the relative abundance of 11 intersection genes (Table 3) involved in significantly enriched lipid metabolism pathways were examined independently by qPCR in chickens with HAbF and LAbF phenotypes. With the exception of caveolin 2 (*CAV2*), the remaining 10 genes showed consistency between the qPCR assays and the deep sequencing analysis in terms of the direction of regulation and statistical significance (Fig. 4).

### Construction of a miRNA-mRNA mediated network

**Figure 5:**
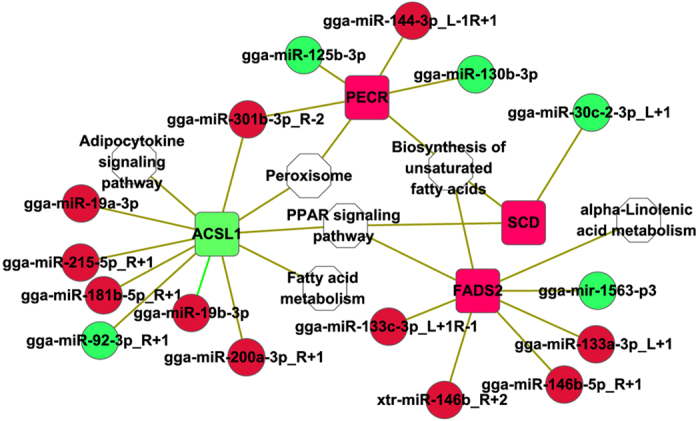
The miRNA-mRNA network between four key intersection genes and differentially expressed miRNAs. Red indicates up-regulation and green indicates down-regulation; white indicates the pathway. *FADS2*: fatty acid desaturase 2; *SCD*: stearoyl-CoA desaturase; *PECR:* peroxisomal trans-2-enoyl-CoA reductase; *ACSL1*: acyl-CoA synthetase long-chain family member 1; gga: *Gallus gallus*; xtr: *Xenopus (Silurana) tropicalis*.

To further understand the function of the intersection genes on abdominal adipose content, specific attention was given to the interaction between the 11 intersection genes involved in significantly enriched pathways and their 34 corresponding DEMs (Table 4). Four of these genes, *ACSL1*, *FADS2*, *SCD*, and *PECR*, were of particular interest because they play roles in multiple pathways. To better visualize and understand the interaction between them and their corresponding DEMs, a miRNA-mRNA network was constructed (Fig. 5).

### Effect of gga-miR-19b-3p on the proliferation of preadipocytes and the differentiation of adipocytes

**Figure 6:**
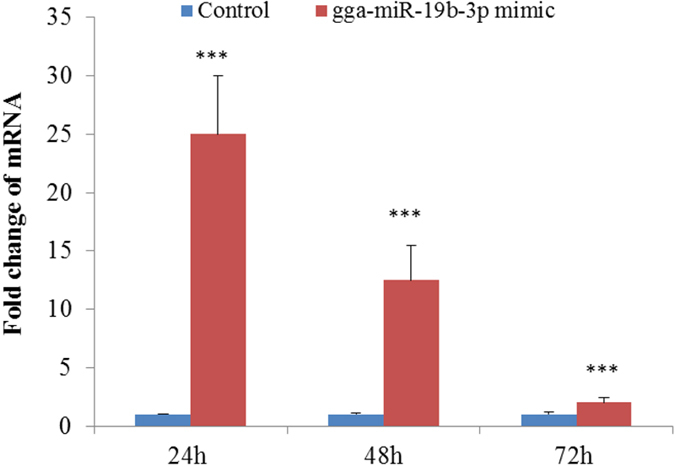
The changes in gga-miR-19b-3p expression after transfection with a gga-miR-19b-3p mimic, ***P < 0.001 vs. the control group.

**Figure 7:**
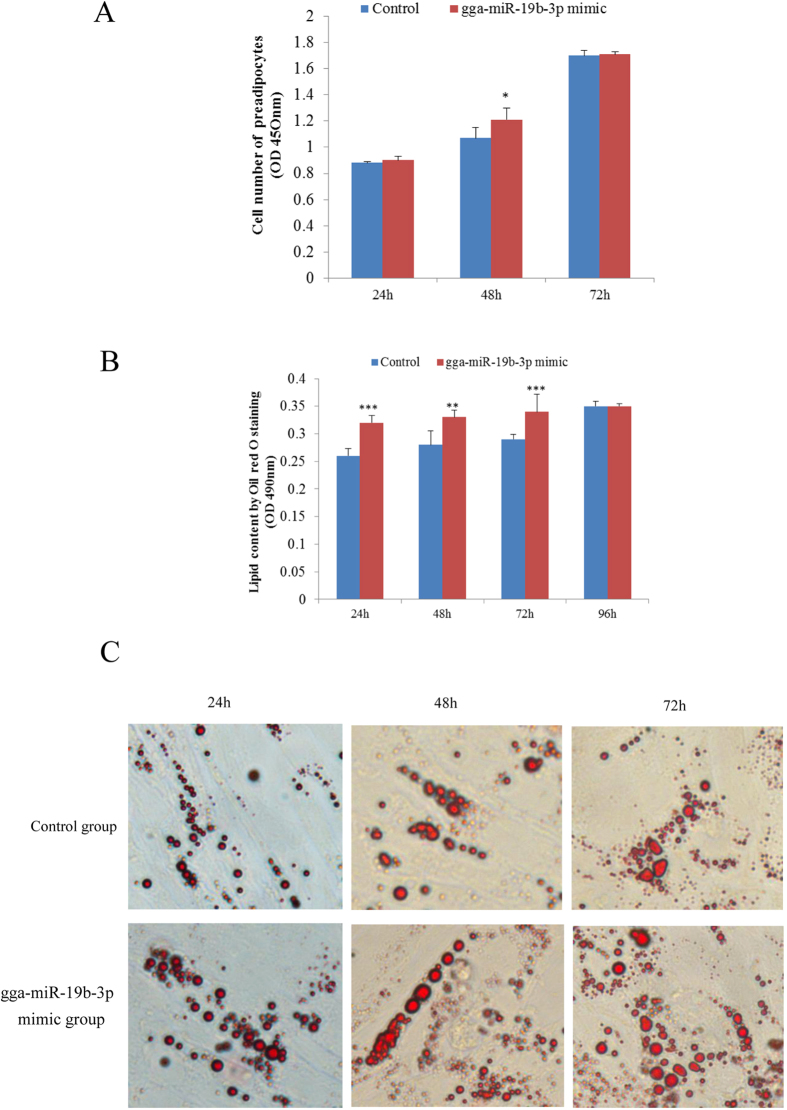
The effect of gga-19b-3p overexpression on the proliferation of preadipocytes and the differentiation of adipocytes, *P < 0.05, **P < 0.01, and ***P < 0.001 vs. the control group. (**A**) the number of preadipocytes was identified using the CCK-8 kit (n = 8), *P < 0.05 vs. the control group. (**B**) Differentiation of adipocytes was identified by Oil Red O staining (n = 3), ***P < 0.01 and ***P < 0.001 vs. the control. (**C**) Morphological changes and lipid deposition induced by 50 nM of the gga-19b-3p mimic in preadipocytes during *in vitro* differentiation (inverted microscope, 400×). lipid droplet, stained with Oil Red O, accumulated as more and larger locules in cells exposed to 50 nM of the gga-19b-3p mimic when compared with those not treated with the gga-19b-3p mimic.

To further explore the biological significance of gga-miR-19b-3p, we observed the effect of gga-miR-19b-3p overexpression on the proliferation of preadipocytes and differentiation of adipocytes in chickens. As shown in Fig. 6, the expression of gga-miR-19b-3p, compared with the negative control, significantly increased after transfection with a gga-miR-19b-3p mimic (*p* < 0.001). When preadipocytes were cultured with 50 nm of the gga-miR-19b-3p mimic, cell numbers increased from 24 to 72 h compared with that of the negative control. At 48 h, the effect of the gga-miR-19b-3p mimic was significant (*p* < 0.05, Fig. 7A). The differentiation of adipocytes significantly increased at 24, 48, and 72 h after transfection with 50 nM of the gga-miR-19b-3p mimic compared with that of the negative control (*p* < 0.01 or *p* < 0.001, Fig. 7B). Oil Red O staining results showed that lipid droplets in cells treated with 50 nM of the gga-miR-19b-3p mimic were slightly larger and more numerous than those of the negative controls (Fig. 7C).

### *ACSL1* is an endogenous target of miR-19b-3p

**Figure 8:**
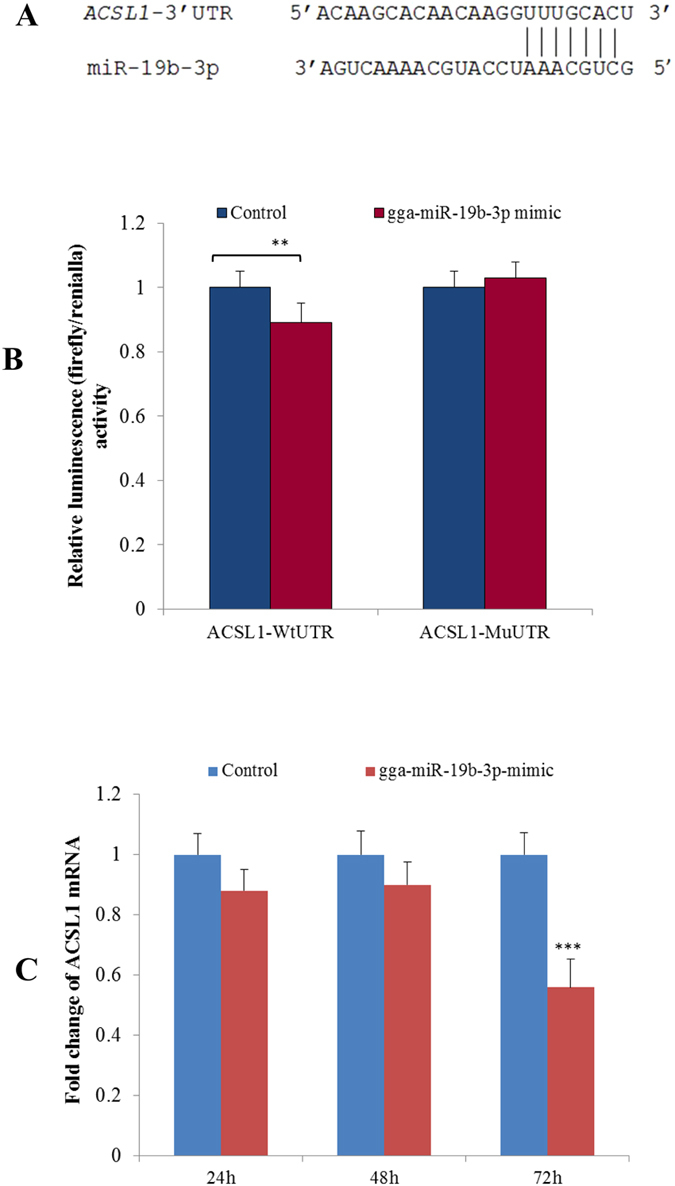
Chicken acyl-CoA synthetase long-chain family member 1 (ACSL1) is a target for gga-miR-19b-3p. (**A**) Nucleotide sequences of the wild-type and mutated gga-miR-19b-3p binding sites located in the 3′ untranslated region (UTR) of *ACSL1*. (**B**) Luciferase activity assay of the wild-type (Wt) or mutant (Mu) 3′ UTR of *ACSL1* using a dual luciferase reporter system in 293T cell lines following co-transfection with the gga-19b-3p or negative control (NC) mimics. Data are derived from five transfectants. (**C**) Changes in *ACSL1* mRNA expression after transfection of the gga-miR-19b-3p mimic (***P < 0.001). Data are derived from triplicate tranfectants.

As shown in Fig. 5, *ACSL1* was down-regulated, while gga-miR-19b-3p was up-regulated, in birds with high AbF. From the analysis using the TargetScan and miRanda algorithms, *ACSL1* transcripts may be a target of miR-19b-3p. This was explored via the co-transfection of luciferase reporter vectors containing the wild-type or mutant 3′ UTR of *ACSL1* (Fig. 8A) and the miR-19b-3p mimic, or a negative control mimic, in 293T cells. As shown in Fig. 8B, the luciferase activities of the wild-type *ACSL1* reporter co-transfected with the miR-19b-3p mimic were reduced significantly compared with that co-transfected with the negative control mimic or in mutant reporters co-transfected with the miR-19b-3p mimic. Compared with the negative control, the level of *ACSL1* mRNA significantly decreased in chicken adipocytes at 72 h after transfection with the miR-19b-3p mimic (Fig. 8C); because anti-ACSL1 antibodies suited for chickens were lacking, changes in the level of the *ACSL1* protein were not measured. Taken together, these results suggest that gga-miR-19b-3p accelerates the proliferation of preadipocytes, as well as adipocyte differentiation, by down- regulating *ACSL1*.

## Discussion

### miRNA and abdominal fat in chicken

In the present study, miRNAs identified by deep sequencing in the HAbF and LAbF libraries from an F2 resource population covered all 48 known miRNAs in chicken abdominal adipose tissue that were identified by cloning^14^. However, many important candidate miRNAs related to lipid mechanism (e.g., gga-miR-301b-3p, gga-miR-130b-3p, gga-miR-30a-5p, gga-miR-142, gga-miR-146b, gga-miR-103, gga -miR-26a, etc.) were missed by cloning. The present work has shown that more candidate miRNAs related to abdominal fat were identified using deep sequencing technology.

Among the miRNAs quantified here, miR-30d and miR-26a were down-regulated in F2 birds with higher AbF content, and these results were confirmed by qPCR in the birds with phenotypic extremes of AbF. Previous work demonstrated that miR-30d and miR26a are highly expressed in chicken preadipocytes^19^ and that miR-30d influences the transcription of insulin^25^. Taken together, these findings indicate that miR-30d and miR-26a are likely to play important regulatory roles in lipid mechanism in chickens.

Changes in four lipid-related DEMs identified by deep sequencing (gga-miR-122-5p, miR-103-3p, miR-27b-3p, and miR-146b-5p) were confirmed by qPCR. For example, the extensive (six- to eight-fold) down-regulation of gga- miR-122-5p was shown by both by deep sequencing and qPCR. In mice, miR-122 is involved in cholesterol and lipid metabolism, as well as in the replication of hepatitis C virus^26^,^27^. The DEM miR-103 was up-regulated, and it regulates glucose homeostasis and insulin sensitivity in obese mice^28^ and up-regulates many marker genes and triglycerides in 3T3-L1 cells^29^. The DEM miR-27 has an inhibitory role with regard to adipogenesis in mouse and human multipotent adipose-derived stem (hMADS) cells^30^, and overexpression of miR-27 specifically inhibited adipocyte formation in mice^31^. miR-146b has been related to the differentiation of 3T3-L1 cells^29^. Although detailed functional characterization of these four miRNAs in chicken AbF is lacking, the present findings, combined with those of previous studies, suggest that these miRNAs may play key roles in the regulation of this tissue in chickens.

The miR17-92 cluster comprises seven miRNAs (miR-17-5p, miR-17-3p, miR-18a, miR-19a, miR-20a, miR-19b, and miR-92-1), and it has been shown to accelerate adipocyte differentiation by negatively regulating the tumor-suppressor Rb2/p130^32^. It is noteworthy that several of these miRNAs (gga-miR-17-5p, gga-miR-19a, gga-miR-19b and gga-miR-20a) were DEMs here (fold change 1.85 to 7.23) and were up-regulated in AbF from birds with high AbF contents. Of these, there was a striking difference (10 fold greater in birds with high AbF) in gga-miR-19b-3p expression, as quantified by qPCR, and the function of this miRNA in AbF is now being further studied.

### Intersection genes and abdominal fat content

This study identified 106 intersection genes, of which 11 are involved in lipid metabolism pathways and clear roles are known for *ACSL1*, *FADS2*, *ABCD3* and *SCD*. Long-chain acyl CoA synthetase (ACSL) activates the breakdown of long-chain fatty acids into acyl-CoA thioesters^33^. *ACSL1* is the main isoform, and its gene is highly expressed in adipose, liver, and muscle in rats^34^,^35^. Additionally, it influenced lipolysis^36^ and β-oxidation rates^37^ in 3T3-L1 adipocytes. The desaturase FADS2 is rate-limiting in the synthesis of long-chain polyunsaturated fatty acids (PUFAs). In pregnant rats, changes in progesterone and estradiol may promote the synthesis of LC PUFA by increasing *FADS2* expression^38^ and epigenetic regulation contributes to the short- and long-term regulation of PUFA synthesis^39^; single nucleotide polymorphisms (SNPs) of *FADS2* affected the content of essential fatty acid in muscle, as well as body weight, in an F2 resource population of Gushi chickens crossed with Adak broilers^40^. *SCD* affected lipid synthesis, lipid oxidation, thermogenesis, and insulin sensitivity in liver, muscle, and adipose tissue in mice^41^, and it may play a potential role in the control of bodyweight and energy homeostasis in chickens^42^. *ABCD3* plays roles in the oxidation of dicarboxylic acids, as well as buffering fatty acids in humans^43^, and it is involved in the regulation of fatty acid transport into peroxisomes in rats^44^.

Among the other intersection genes, laminin, alpha 2 (*LAMA2*) and alpha 11 integrins (*ITGA11*) and *PECR*, while known in other contexts^45^,^46^, have no obvious relationship to fat.

No previous studies have associated *ACSL1*, *FADS2* and *ABCD3* with lipid metabolism, specifically in chickens. On the grounds that they displayed significant differential expression here, further studies of these genes seem to be warranted. Additional genes identified here include *CHAD*, *AKT1*, and *LAMA2*. If or how they affect lipid metabolism or fat accumulation is not apparent; thus, these genes require further study in chicken AbF.

In this study, some of the DEMs relevant to the 11 intersection genes may be strong candidates for regulating AbF because miR-133c-3p, miR-133a-3p, miR-200a-3p, and miR-146b-5p were shown by independent qPCRs to be differentially expressed in birds with very high and very low AbF contents. Some of the DEMs identified by deep sequencing (miR-19a-3p, miR-19b-3p, miR-30d, miR-26a, miR-30a-5p, miR-122-5p, miR-103, miR-125b, and miR-17-5p) are known to influence mammalian lipid metabolism. To demonstrate the applicability of the approach used in this study, one of the DEMs, gga-miR-19b-3p, significantly increased the proliferation of preadipocytes, as well as adipocyte differentiation, after transfection with 50 nM of a gga-miR-19b-3p mimic, compared with that of negative control. Additionally, it was shown here by a luciferase reporter assay to clearly down-regulate *ACSL1* expression. When the adipocytes were treated with 50 nm of a gga-miR-19b-3p mimic, *ACSL1* mRNA expression was significantly reduced compared with that of a negative control. These results indicate that gga-miR-19b-3p contributes to the increased accumulation of AbF by down-regulating *ACSL1* in chickens. The results obtained here will guide the comprehensive, functional study of the DEMs and intersection genes in regulating the quantity of AbF accumulated in growing chickens.

In conclusion, differences were demonstrated in the transcriptome and miRNA of the AbF of chickens with high and low AbF contents. Specifically, the integrated analysis of DEMs and DEGs suggests that nine miRNAs (gga-miR-19a-3p, miR-19b-3p, miR-17-5p, miR-30d, miR-26a, miR-103-3p, miR-27b-3p, miR-142-3p, and miR-92-3p) and three genes (*ACSL1*, *FADS2* and *ABCD3*) are strong candidate miRNAs and genes involved in regulating the accumulation of AbF in chickens. Potentially novel miRNAs (gga-miR-3535, miR-30e-5p, miR-301b-3p, miR-215-5p, miR-200a-3p, miR-133a-3p, miR-133c-3p, and miR-146b-5p) and genes (*LAMA2, RAP1B, PECR, AKT1, ITGALL and CHAD*) related to abdominal adipose tissue were also identified.

## Material and Methods

### Ethics Statement

The methods of this study were conducted in accordance with the Guidelines for Experimental Animals established by the Ministry of Science and Technology (Beijing, China). All experimental protocols were approved by the Science Research Department (in charge of animal welfare) of the Institute of Animal Sciences, Chinese Academy of Agricultural Sciences (CAAS) (Beijing, China).

### Experimental animals

The CAAS chicken F2 resource population was used. The population, already described in detail^47^, was derived from crosses between a slow-growing Chinese local breed (Beijing-You) and a rapid-growing commercial broiler line (Cobb-Vantress, Inc.).

The chickens were raised in stair-step cages under the same recommended environmental and nutritional conditions at the conservation farm of the Institute of Animal Sciences (IAS), CAAS.

Chickens were weighed and killed by stunning and exsanguination at 93 days of age, 12 h after feed was withheld. Samples of abdominal fat tissue were snap-frozen in liquid nitrogen, then held at −80 °C. During dissection, AbFW was measured and AbFP, relative to the eviscerated weight, was calculated. Based on these values for 183 females, two phenotypic groups, each comprising six birds with high AbF (HAbF) or low AbF (LAbF) were assembled. Threshold values for the HAbF group were AbFW ≥ 66.64 g and AbFP ≥ 6.49%, and those for the LAbF group were AbFW ≤ 34.50g and AbFP ≤ 1.84%, representing means + standard deviation (SD) for the population studied. Individual data for the two cohorts are given in Table 5.

### RNA extraction

Total RNA was isolated from abdominal fat tissue using a commercially available kit according to the manufacturer’s protocol (DP419, Tiangen, Beijing, China), Trizol was from Invitrogen (Carlsbad, CA, USA). The concentration and purity of RNAs were determined by A_260_ and A_260:280_ (A_260:280_ ≥ 1.8 and ≤2.0) using a NanoDrop ND-1000 spectrophotometer (Nanodrop Technologies, Wilmington, DE, USA). RNA integrity (RIN ≥ 7 and 28S/18S ≥ 0.7) was assessed on an Agilent 2100 Bioanalyzer Lab-on-chip system (Agilent Technologies, Palo Alto, CA, USA). RNA samples were stored at −80°C until used.

### Construction and sequencing of small RNA libraries

Two equal pools of total RNA, each from six chickens (500 ng per sample), were generated from the HAbF and LAbF birds (Table 5). Two small RNA libraries were prepared according to Illumina’s instructions and sequenced (Illumina GAIIx, Illumina, San Diego, CA, USA). Raw sequencing reads, obtained with Illumina’s Pipeline v1.5 software, were filtered to obtain mappable sequences using ACGT101-4.2 (LC Sciences, Hangzhou, China) based on mammalian data in miRBase 19.0. Modified reads per million reads (RPM) was used to quantify the normalized reads.

### mRNA sequencing

Pools from the same birds (4 μg/sample) were used for mRNA sequencing (LC Sciences), and raw data were obtained using CASAVA v1.8+ (Illumina). Sequences were aligned to the chicken genome using TopHat. Sequence segments were spliced, annotated, and transcript expression was calculated by Cufflinks. Fragments per kilobase of exon per million mapped reads (FPKM) was employed to quantify gene expression and the Ensemble database was used as a reference.

### Bioinformatic analyses

The chicken (*Gallus gallus*) Ensemble database, targetScan and the miRanda algorithm were employed to predict potential targets of all the differentially expressed miRNAs. Pathways and GO classification were analyzed using Kobas2.0 (http://kobas.cbi.pku.edu.cn/help.do) and hypothesis testing. The significant pathways were intersection between Kobas2.0 and hypothesis testing method. *P* ≤ 0.05 was considered to be significant. Pathways and GO terms with less than three known chicken genes were discarded. The miRNA-mRNA interaction network was constructed using Cytoscape software.

### Quantitative real-time PCR of mRNA and miRNA

The relative transcript abundances of 21 differentially regulated genes, identified by mRNA sequencing, were independently validated using the Quantifast SYBR Green PCR Kit (Qiagen, Düsseldorf, Germany). The final concentration of each primer was 10 μmol/μL. The primers used are described in Table S6.

To increase the power of this test, AbF samples from distinct birds were used, again consisting of six HAbF and six LAbF individuals; they were Beijing-You hens at 93 days of age, as further described in Table 6.

Parallel quantitative real-time PCRs (qPCRs) were used to quantify relevant miRNAs, also using Qiagen’s methodology (the miScript II RT Kit and the miScript SYBR Green PCR Kit) with a miRNA-specific forward primer (Table S7 and the universal reverse primer provided). U6 was chosen as an internal control to correct for analytical variations. The concentration of each primer was 10 μmol/μL. Differences between the two groups were analyzed using Student’s t-tests for independent samples in SAS 8.0 for Windows (SAS Inst. Cary, NC, USA).

### Vector Construction

The 3′ UTR of *ACSL1* containing a miR-19b-3p binding site was amplified from chicken genomic DNA by PCR with the primers shown in Table S6. PCR products were cloned into psiCHECK-2 (Promega, Madison, WI, USA) using the NotI and XhoI restriction sites. Mutant target vectors, which had a 7 bp substitution in the binding site (TTTGCAC → AAACGTG) were obtained from the Ribo Company (Guangzhou, China).

### Luciferase reporter assays

Luciferase reporter experiments were performed in human embryonic kidney (HEK) 293T cells. Cells were seeded in 96-well plates at a density of 5 × 10^4^ cells/well and cultured under routine conditions with 10% fetal bovine serum. When the cells reached 60% to 70% confluence, pmirGLO-3′ UTR (100 ng) was co-transfected with 50 nM of a negative control or a gga-miR-19b-3p mimic (both from Ribo) using 0.25 μL of FugeneHD (Promega) according to the manufacturer’s instructions. The relative luciferase activity was measured 48 h after transfection by the Dual-Glo Luciferase Assay System (Promega).

### Cell proliferation and differentiation assay

Preadipocytes isolated from abdominal adipose tissue from 2 to 4-week-old female Beijing-You chickens, following published methods^48^,^49^, were seeded in six-well plates. Once the cells reached 70% confluence, differentiation was induced with MDI (IBMX, 0.5 mmol/L, DEX, 1 μmol/L, and insulin, 1 mg/L). After 24 h, cells were transfected with a gga-miR-19b-3p mimic (50 nM) or a negative control (50 nM) using 6 μL of FugeneHD. Subsequently, cells were fixed with 10% formaldehyde at 24, 48, and 72 h, washed with phosphate-buffered saline (PBS), stained with Oil Red O (0.3% in 60% isopropanol), followed by extensive washes, and the stained triglyceride droplets were visualized and photographed. The transfection was performed in triplicate. Cells of the same group in six-well plates transfected with the gga-miR-19b-3p mimic were harvested and total RNA was extracted to identify the differential expression of ACSL1 mRNA. The effects of overexpressing gga-miR-19b-3p on preadipocyte proliferation were assessed using the Cell Counting Kit-8 (CCK-8, Dojindo Molecular Technologies, Kumamoto, Japan) at 24, 48, and 72 h.

## Additional Information

**How to cite this article**: Huang, H. Y. *et al.* Integrated analysis of microRNA and mRNA expression profiles in abdominal adipose tissues in chickens. *Sci. Rep.* **5**, 16132; doi: 10.1038/srep16132 (2015).

## Supplementary Material

Supplementary Information

Supplementary Table S1

Supplementary Table S2

Supplementary Table S4

Supplementary Table S5

## Figures and Tables

**Table 1:** Six categories of generated miRNA sequences.

Category	Group		Sequence
Reads mapped to selected pre-miRNAs in miRbase	Group 1	Group 1a	Mapped to *Gallus gallus* known pre-miRNAs
		Group 1b	Mapped to mammalian known pre-miRNAs which could be mapped to *Gallus gallus* genome
	Group 2	Group 2a	Mapped to mammalian known pre-miRNAs which could not be mapped *Gallus gallus* genome, but reads mapped to Gallus gallus genome and within hairpins
		Group 2b	Mapped to mammalian known pre-miRNAs which unmapped to *Gallus gallus* genome, but reads mapped to Gallus gallus genome, no hairpins.
	Group3	Group 3a	Mapped to mammalian known pre-miRNAs which unmapped to *Gallus gallus* genome, reads unmapped to Gallus gallus genome, but reads mapped to mature miRNAs.
		Group 3b	Mapped to mammalian known pre-miRNAs which unmapped to *Gallus gallus* genome, reads unmapped to Gallus gallus genome, but reads unmapped to mature miRNAs.
Reads unmapped to selected mirs in miRbase	Group4	Group 4a	Unmapped to known miRNAs but mapped to *Gallus gallus* genome and within hairpins
		Group 4b	Unmapped to known miRNAs but mapped to *Gallus gallus* genome and without hairpins
	Group 5	others	Mapped to other defined databases, such as mRNA, RFam, or Repbase
	Group6	No hit	None of the above

**Table 2:** Significantly enriched pathways for intersection genes

Pathway name	Enriched genes	*p-*value
Fatty acid metabolism	*ACSL1, FADS2, SCD, PECR*	0.00
Biosynthesis of unsaturated fatty acids	*FADS2, SCD, PECR*	0.00
Focal adhesion	*ITGA11, RAP1B, CHAD, CAV2, LAMA2, AKT1*	0.01
PPAR signaling	*FADS2, SCD, ACSL1*	0.02
Peroxisome	*ACSL1, ABCD3, PECR*	0.03
ECM-receptor interaction	*LAMA2, ITGA11, CHAD*	0.03

**Table 3:** Intersection genes involved in lipid metabolism pathways.

Gene Abbreviation	Gene Describle	Fold value*	*p*-value
*SCD*	stearoyl-CoA desaturase (delta-9-desaturase)	5.25	0.00
*CAV2*	caveolin 2	2.91	0.00
*PECR*	peroxisomal trans-2-enoyl-CoA reductase	2.77	0.01
*FADS2*	fatty acid desaturase 2	2.40	0.01
*ABCD3*	ATP-binding cassette, sub-family D (ALD), member 3	2.70	0.01
*AKT1*	v-akt murine thymoma viral oncogene homolog 1	2.04	0.02
*RAP1B*	RAP1B, member of RAS oncogene family	2.03	0.03
*ITGA11*	integrin, alpha 11	0.43	0.01
*LAMA2*	laminin, alpha 2	0.33	0.00
*CHAD*	chondroadherin	0.32	0.00
*ACSL1*	acyl-CoA synthetase long-chain family member 1	0.30	0.00

^*^Relative mRNA abundance of the gene when comparing the performance in the high abdominal fat library to that in the low abdominal fat library sequenced by deep sequencing.

**Table 4:** Eleven intersection genes and the corresponding differentially expressed miRNAs*.

Differentially expressed genes	miRNA	Regulation direction of miRNA
*ABCD3* (up)	gga-miR-30d_R + 2	down
	gga-miR-181b-5p_R + 1	up
	gga-miR-19a-3p	up
	gga-miR-19b-3p	up
*ACSL1* (down)	gga-miR-200a-3p_R + 1	up
	gga-miR-215-5p_R + 1	up
	gga-miR-301b-3p_R-2	up
	gga-miR-92-3p_R + 1	down
	gga-miR-133a-3p_L + 1	up
	gga-miR-133c-3p_L + 1R-1	up
	gga-miR-146b-5p_R + 1	up
*FADS2* (up)	gga-mir-1563-p3	down
	gga-miR-204	up
	gga-mir-1595-p5_1ss16GC	down
	gga-miR-30a-3p	down
	gga-miR-30c-2-3p_L + 1	down
	gga-miR-200a-3p_R + 1	up
	gga-miR-30a-5p_R + 2	up
	gga-miR-30c-5p	up
	gga-miR-30e-5p_R + 5	up
*SCD* (up)	oan-miR-143-5p_L-1R + 1	up
	gga-miR-1563_R + 6_1ss13GA	down
	gga-miR-23b-3p	down
	gga-miR-27b-3p	down
	gga-miR-30d_R + 2	down
	gga-miR-144-3p_L-1R + 1	up
	gga-miR-301b-3p_R-2	up
*PECR* (up)	gga-miR-125b-3p	down
	gga-miR-130b-3p	down
	gga-miR-26a-5p_R + 1	down
*AKT1* (up)	gga-mir-1563-p3	down
	gga-miR-92-3p_R + 1	down
	gga-miR-144-3p_L-1R + 1	up
	gga-miR-200a-3p_R + 1	up
	gga-miR-103-3p	down
	gga-miR-107-3p_R-1	down
	gga-miR-125b-3p	down
	gga-miR-128-3p	down
*CAV2* (up)	gga-miR-128-3p	down
*CHAD* (down)	gga-miR-30c-1-3p_R + 1	down
	tgu-miR-456-3p_R + 1	down
*ITGA11* (down)	gga-mir-1595-p5_1ss16GC	down
*LAMA2* (down)	gga-miR-30c-1-3p_R + 1	down
	gga-miR-30c-2-3p_L + 1	down
*RAP1B* (up)	gga-miR-181b-5p_R + 1	up
	gga-miR-19a-3p	up
	gga-miR-19b-3p	up
	gga-miR-200b-3p	up
	gga-miR-204	up
	gga-miR-301b-3p_R-2	up
	gga-miR-30a-5p_R + 2	up
	gga-miR-30c-5p	up
	gga-miR-30e-5p_R + 5	up
	gga-miR-7b_R + 3	up
	gga-miR-128-3p	down
	gga-miR-130b-3p	down
	gga-miR-1563_R + 6_1ss13GA	down
	gga-miR-26a-5p_R + 1	down
	gga-miR-27b-3p	down
	gga-miR-30d_R + 2	down

^*^Regulation direction when comparing the performance of the gene or miRNA in the high abdominal fat library to that in the low abdominal fat library sequenced by deep sequencing.

**Table 5:** Phenotypic differences of the birds from the F2 resource population used to create pools for miRNA and mRNA deep sequencing*.

High group			Low group		
Sample	AbFW (g)	AbFP (%)	Sample	AbFW (g)	AbFP (%)
H-1	90.38	5.28	L-1	25.60	1.66
H-2	119.90	5.79	L-2	23.00	1.72
H-3	107.04	5.85	L-3	26.22	1.84
H-4	72.65	5.15	L-4	23.28	1.79
H-5	87.30	5.62	L-5	18.90	1.44
H-6	66.64	6.49	L-6	34.50	1.80
Mean ± SD	90.65 ± 20.17**	5.70 ± 0.48**	Mean ± SD	25.25 ± 5.21	1.71 ± 0.14

^*^AbFW: abdominal fat weight; AbFP: percentage of AbFW to eviscerated weight.

^**^P < 0.01 when compared with the mean in the Low group.

**Table 6:** Phenotypic differences between Beijing-You chickens used for qPCR validation of differentially expressed miRNAs and genes*.

Traits	Group	
	Low	High
AbFW (g)	10.81 ± 1.43	65.99 ± 7.56**
AbFP (%)	1.46 ± 0.31	7.33 ± 0.88**

^*^AbFW: abdominal fat weight; AbFP: percentage of AbFW to eviscerated weight; the High group (n = 6) consisted of samples from chickens with the highest trait values, and the Low group (n = 6) consisted of samples from chickens with the lowest trait values.

^**^P < 0.01 when compared with the mean in the Low group.
