# Supplementary material for: Integrated analysis of microRNA and mRNA expression profiles in abdominal adipose tissues in chickens
Source: Sci Rep. 2015 Nov 4;5:16132. doi: 10.1038/srep16132 (PMC4632014; doi:10.1038/srep16132)
Supplement: Supplementary Information [file srep16132-s1.pdf]

Supplementary information file

Integrated analysis of microRNA and mRNA expression profiles in abdominal adipose tissues in chickens

Huang, H.Y.[1,2#], Liu, R.R.[1#], Zhao, G.P[1], Li, Q.H[1], Zheng, M.Q[1], Zhang, J.J[1], Li, S.F[2], Liang, Z[2], Wen, J[1*]

[1]Institute of Animal Sciences, Chinese Academy of Agricultural Sciences, Beijing 100193, P.R. China;

[2] Institute of Poultry Science, Chinese Academy of Agriculture Sciences, Yangzhou 225125, P. R. China;

[#] These authors contributed equally to this work.

Supplemental Figure Legends

Figure S1. Length distribution of small RNAs. LAbF: low-abdominal fat libraries; HAbF: high-abdominal fat libraries.

Figure S2. Exonic rates of HAbF and LAbF libraries regarding the mRNA-seq experiments. (A) Exonic rates of LAbF group (B) Exonic rates of LAbF group; LAbF: low-abdominal fat libraries; HAbF: high-abdominal fat libraries.

Figure S1

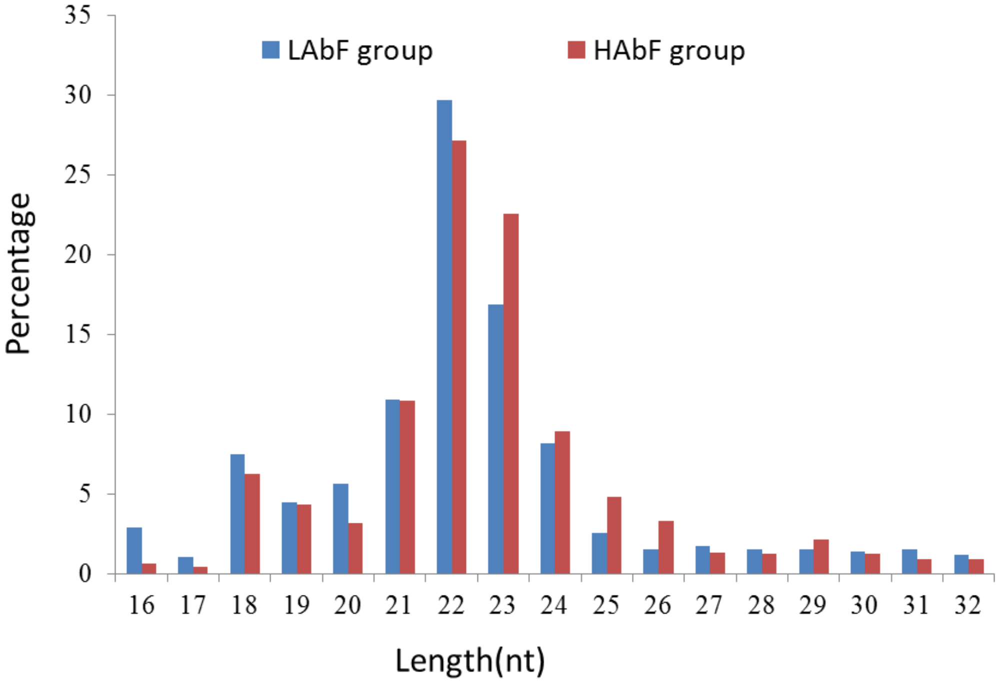
LAbF group
HAbF group
Percentage
Length(nt)
35
30
25
20
15
10
5
0
16
17
18
19
20
21
22
23
24
25
26
27
28
29
30
31
32

Figure S2

LAbF: 66424 HAbF: 63618

A

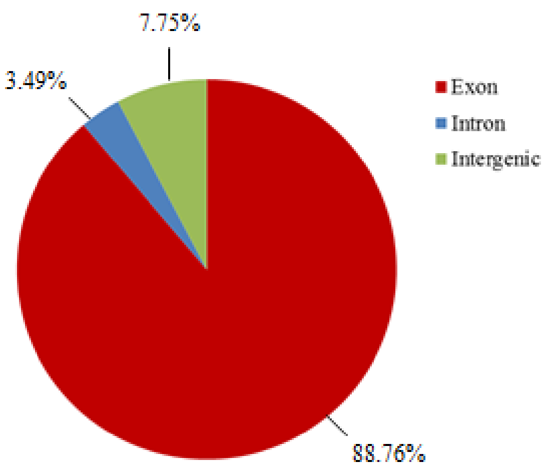

B

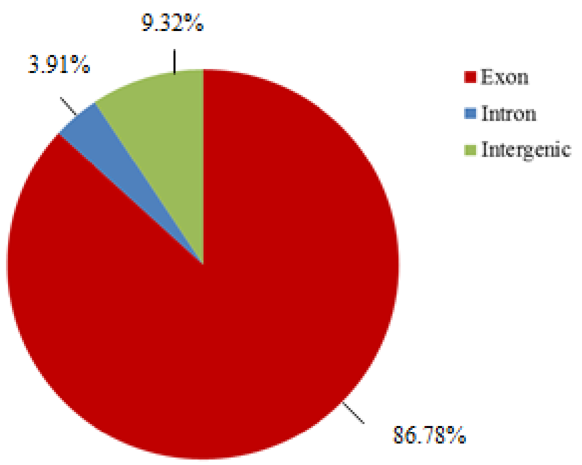

Table S3. Produced Reads and Aligned Reads of HAbF and LAbF libraries regarding the mRNA-seq experiments

		LAbF	Valid Ratio (reads)%	HAbF	Valid Ratio(reads)%
Produced Reads	Raw Reads	49242752	100%	57429740	100%
	Valid Reads	49242752		57429740	
Aligned Reads	Total Valid reads	49226144	100.00%	57351996	100.00%
	Read-1	8552400	17.37%	11029633	19.23%
	Read-2	8340873	16.94%	10814590	18.86%
	Alignment	16893273	34.32%	21844223	38.09%
	Unmapped	32332871	65.68%	35507773	61.91%

LAbF: low-abdominal fat libraries

HAbF: high-abdominal fat libraries

Alignment: The total number of reads mapped to the reference genome

Unmapped: The total number of reads that were not mapped to the reference genome

Table S6. q-PCR primers for genes

Gene name	Accession Number	Sequence (5' to 3')
AKR1D1	NM_001277393.1	F: GGATGAAACGTGGGTAAATG
		R: CTCTGATGCGTTGTGGATTG
APOA5	XM_417939.4	F: AGCAGATCATGTCGCTGAAG
		R: TTCCTTCAGATTTGTGAACTC
CYP11A1	NM_001001756.1	F: TTCCTTCAGATTTGTGAACTC
		R: CACAGAATGCTTCCTTGTGT
SOCS3	NM_204600.1	F: CCTCTGCCGTAAGACTGTCA
		R: TTTCCCTCTGCCAGCCTCTT
APOA1	NM_205525.4	F: GGACCAGTTCTCCGCCAAGT
		R: TGCACCAGAGGCGTCATC
SCP2	XM_004936799.1	F: TAAGCGAAGGAATGAAAC
		R: AGAAGATATGGGTAGCAC
CTBP1	NM_001012890.1	F: CTTTGCTTTCAGCCGTCAC
		R: ATTGGTCGCTTGGGTGGT
PRKAR2B	XM_001232696.3	F: ATGGACATTGCCGACACC
		R: GGTCTTCATTTCAGGTGG
PLTP	NM_001162406.1	F: TTCTGCTGATGGTGTCCACATC
		R: GGAACTCATAGACCTTCCTGAG
FADS2	NM_001160428.2	F: TCCTCACTTTTGTCAGGTTTCT
		R: CTGAGAGCTAAGCCAGTCTTTG
PECR	NM_001006522.1	F: AACAGTGTTGCTCCTGGAATAG
		R: ATCTCCTCAGGAACTGCTGATC
PTGES	NM_001194983.1	F: GCTCAGGAAGAAGGCATTTG
		R: TACACAGCTCCAAGGAAGAG
SCD	NM_204890.1	F: TGCTACAGGAGGAGGAGTTCT
		R: CTTCTCACGGTAGGTTTCATC
ABCD3	NM_001012597.1	F:CGTGTTCTTGGTGAGTTGTG
		R: TTCCTTCAGCACCTGGTCAGG
ACSL1	NM_001012578.1	F: CAAAGGAGAAGGTGAGGTGTG
		R: CTTCAACGTACCGTTTGGTAG
LAMA2	XM_419746.4	F: CCAGGAATGTGAGTGTGATCAATAT

		R: AGAAGTCTCTCTGGTGCTCGCT
CHAD	XM_004946335.1	F: TCCTGCTTCTTCTCTTCCCACCACA
		R: GTTTGCTCAGGCACCTTGGGGAT
GART	NM_001001469.1	F: TTCTCAAGTTGGCGTCTCTGGTGAT
		R: TGTGTGCATAAGCTTTCACGTGGC
AKT1	NM_205055.1	F: TGGCACATTCATTGGCTAC
		R: CTCCAGCATCAAACCTAC
RAP1B	NM_001007852.1	F: GGTTCTTGGTTCTGGAGGTGTT
		R: ATCATCAGTGTCTTTGACTCGAAGA
ITGA11	XM_413930.4	F: AAGGATGCCTATTTTGGCTACA
		R: ACAGAGTGACTCTTCCTAGGTTGAG
CAV2	NM_001007086.1	F: CGCATCTTCATGGACGACGACAAC
		R: GCAGATCCACACCTTGTCGAAGGAG
ACSL1 3’UTR	NM_001012578.1	F:CAACTCGAGACAGTGGATTGAGTAAAGTGG
		R:TAAGCGGCCGCATGCAGAAATATTTATTTTG

Table S7. miRNA-specific primers for q-PCR*

miRNA	Primers
gga-miR-19a-3p	TGTGCAAATCTATGCAAAACTGA
gga-miR-19b-3p	TGTGCAAATCCATGCAAAACTGA
gga-miR-17-5p	CAAAGTGCTTACAGTGCAGGTAGT
gga-miR-30e-5p	TTTCAGTCGGATGTTTACAGC
gga-miR-103-3p	AGCAGCATTGTACAGGGCTATGA
gga-miR-27b-3p	TTCACAGTGGCTAAGTTCTGC
gga-miR-26a-5p	TTCAAGTAATCCAGGATAGGC
gga-miR-30d	TGTAAACATCCCCGACTGGAAG
gga-miR-142-3P	TGTGCAAATCCATGCAAAACTGA
gga-miR-122-5P	TGGAGTGTGACAATGGUGTTTGT
gga-miR-3535	GGATATGATGACTGATTATCTGAAA
gga-miR-301b-3p	CAGTGCAATAGTATTGTCAAAGCAT
gga-miR-133c-3p	TTGGTCCCCTTCAACCAGCTGC
gga-miR-146b-5p	TGAGAACTGAATTCCATAGGCG
gga-miR-215-5p	ATGACCTATGAATTGACAGAC
gga-miR-200a-3p	TAACACTGTCTGGTAACGATGT
gga-miR-92-3p	TATTGCACTTGTCCCGGCCTG
gga-miR-133a-3p	TTGGTCCCCTTCAACCAGCTGT
gga-miR-204	TTCCCTTTGTCATCCTATGCCT
gga-miR-144-3p	CTACAGTATAGATGATGTACTC
U6	GTGACCCACGATGTGTATTCGC
